# Bringing Real-World Microbiology Experiences to Undergraduate Students in Resource-Limited Environments

**DOI:** 10.3389/fmicb.2020.589405

**Published:** 2020-12-08

**Authors:** Y. Aparna, K. Anuradha, Ch. Jyothi, K. Sri Manjari, Anil Kumar Challa

**Affiliations:** ^1^Bhavan’s Vivekananda College of Science Humanities and Commerce, Secunderabad, India; ^2^St.Ann’s College for Women, Hyderabad, India; ^3^University College for Women, Osmania University, Hyderabad, India; ^4^Dr. Reddy’s Institute of Life Sciences, Hyderabad, India; ^5^Department of Biology, The University of Alabama at Birmingham, Birmingham, AL, United States

**Keywords:** microbial diversity, undergraduate academic success, resource limitations, faculty professional development, student workshops, primary research articles

## Abstract

Undergraduate microbiology curriculum should be amenable to periodic changes to incorporate new developments and ideas. The curriculum should be used not merely as a way to disseminate facts but also as a way to allow students to experience the process of science. In the context of undergraduate microbiology education in Osmania University (Hyderabad, India), existing curriculum does not explicitly allow students to engage in deeper understanding of concepts and understanding of the process of science, both in lecture and laboratory courses. The assessment methods that are currently used are limited in scope as they only test factual recall and superficial understanding of the subject and very minimally assess critical thinking skills. Another factor hampering innovation in the broader context of undergraduate education is the unavailability and inaccessibility to adequate resources. To address the issue of resource-limitations in implementing activities that expose undergraduate students to real-world microbiology experiences, a collaboration between a research institute and two teaching colleges was formed. This collaboration involved teacher and student workshops on exploring microbial diversity using 16S rRNA analysis with a view of blending novel research questions with technical skills in the undergraduate microbiology lab. This effort is an example of educators providing students with authentic experiences and, helping them gain critical knowledge and research skills in microbiology even under resource constraints, and students demonstrating motivation to participate in similar activities in the future. The collaborative effort described here can be a broadly sustainable model to improve overall undergraduate education in relatively resource-limited environments.

## Introduction

The goals of undergraduate biology education can range from enabling students to gain broad knowledge and experiences that will prepare them to be become socially conscious citizens who can effectively contribute to the needs of the community (Ferren and Anderson, 2016; Hatcher, 2011; Association of American Colleges and Universities, 2007; Penn, 2011; Zai, 2015). Learning spaces should be engaging, enriching and empowering in order to help students achieve these goals. Real world experiences are inherently rich in context, have the power to engage and are relevant. Bringing these real-world experiences into classrooms can significantly improve the quality of teaching and learning (Alberts, 2005).

Basic science education in India at the undergraduate level, unlike technical and professional education, predominantly happens not on University campuses but in over 10,000 colleges affiliated to degree granting Universities under the purview of the University Grants Commission (Government of India and University Grants Commission [UGC], 2019). With a greater emphasis on didactic classroom instruction, and constrained by resource limitations, it is believed that most of these colleges are unable to provide adequate training to their students to meet the needs of academia and industry. In addition, existing problems with the current higher education system in India pointed by Saberwal (2019), pose a challenge to the introduction of newer ideas and methods, especially in biology education. In a meeting report on “Policy framework for catalyzing excellence in science education and research in India” (Lakhotia et al., 2013), the authors state that “…a rejuvenation of the existing undergraduate and postgraduate science education system together with an integration of teaching with high-quality research is also desperately needed” and point to a steady decline of the infrastructure, quality of faculty and the research capacity of the higher educational system as a whole for over three decades. One of their specific recommendations for improving the current state of undergraduate education is to recognize good teaching practices and development of open-ended laboratory work/research projects by students.

While a top down approach via policy statements and national plans have their role in transforming undergraduate education, true transformation is only possible when individual colleges and informed teachers are empowered to take action. Under the purview of University Grants Commission of India (UGC), which is charged with coordination, determination and maintenance of standards of higher education in India, out of all educational institutions in India, about 750 colleges are autonomous allowing them to design and implement small courses or programs by themselves and supplement the University prescribed syllabus. This autonomy allows administrators and teachers the freedom to experiment and pursue alternative pedagogies to develop innovative and exemplary teaching practices and create rich open-ended learning experiences for students.

While extended research internships in an apprentice model are highly desired to provide rich and comprehensive experiences to students, they are not always possible and scalable due to resource constraints. Studies show that well designed shorter experiences can have equally enriching experiences for students (Wei and Woodin, 2011; Frantz et al., 2017). Here, we describe a program developed through a collaboration between a research institute and teachers at two private autonomous colleges to expose undergraduate students majoring in microbiology to real-world microbiology experiences in Hyderabad, India.

## Professional Development Program – Catalyst for Transformation

A series of teacher (faculty development) workshops developed by the Center for Advancement of Research Skills (CARS) at Dr. Reddy’s Institute of Life Sciences (Hyderabad, India) initiated a dialog between its researchers and undergraduate educators in the local colleges to create specific interventions that would result in changes with respect to student engagement and learning outcomes in college classrooms. Typically, faculty professional development workshops are focused either on discipline-specific content or exclusively on pedagogy. The CARS workshop series made a deliberate attempt to blende these two aspects and placed emphasis on teachers sharing their skill and experiences with their students. The workshops, which were conducted at the research institute, involved teachers assuming student personas to experience a research project while discussing ideas in pedagogy and potential challenges in classroom implementation. The final aspect of the workshops was to create classroom modules or co-curricular activities that they could implement in their respective institutions. A 3-day hands-on workshop on “Exploring Microbial Diversity” was organized for educators with an explicit goal of enabling them to plan and implement student programs that align closely to the existing microbiology curriculum in their respective colleges. Subsequent to the workshop, the research institute provided continuous support to the teachers, as needed, throughout the duration of the student programs in the colleges.

## Classroom Implementation

The current undergraduate degree programs in the sciences are offered as 3-year programs in India. Students opt for specific combinations of subjects upon enrolling into college and continue with the same combination for 3 years. For example, students interested in microbiology can select combinations such as Microbiology, Botany, Chemistry (MBC) or Microbiology, Biochemistry and Chemistry (MBiC), Microbiology, Genetics, Chemistry (MGC), or Microbiology, Zoology, Chemistry (MZC). The syllabus for the courses taught in the above programs is framed and prescribed by the Boards of Studies at the degree-granting University. While University constituents and affiliated colleges are mandated to strictly follow these syllabi, the autonomous colleges (which are a focus of this study) have some flexibility in modifying their curricula and syllabi. This also includes the control of 20–30% of student grades within the college, through internal assessments. This autonomy allowed us the freedom to – design and implement the “Exploring Microbial Diversity” workshop for undergraduate students in two colleges.

Following the teacher workshop, we designed a laboratory workflow that is likely to succeed during classroom implementation, keeping in view the academic semester schedules, alignment with the syllabus and available material resources in the colleges. There were differences between the two colleges that resulted in unique challenges for classroom implementation. Apart from structural constraints, there were inclusivity issues with respect to student enrollments into the workshop. Since the activities would be conducted beyond the official college hours, only those students who could and chose to stay beyond regular class time could enroll in the workshop (Table 1). With regards to the instructional team, two undergraduate instructors from one college who participated in the teacher workshop trained three additional instructors at their own institution to strengthen the program with adequate resources by all means. All the five instructors who were conversant with the laboratory workflow conducted a 3-day trial run at the college to test and ensure proper functioning of chemicals, reagents and equipment. Critical reagents and consumables were supplied by the research institute while the colleges provided the basic laboratory infrastructure. With respect to instruments, micropipette sets, a miniPCR^TM^ thermocycler, a blueGel^TM^ gel electrophoresis unit and a PCR microcentrifuge were made available on loan from the research institute. They were extensively used in one college while the other college did not need all of them to run the workshops.

**TABLE 1:** Outline of the collaborative program development in two phases.

	Phase I	Phase 2		
			College 1	College 2
		Microbiology majors	100 × 3 years = 300	90 × 3 years = 270
		Women	154 (51%)	270 (100%)
		Men	146 (48.6%)	0 (0%)
	Exploring Microbial Diversity by 16S rRNA profiling			
Activity	Teacher workshop		Student workshop	Student workshop
Organizer	Research Scientist		College Teachers (5)	Research Scientist + Teaching Assistant
Venue	Research Institute		Bhavan’s Vivekananda College	St. Ann’s College for Women
Participants	College Teachers (4)		BSc II year students (30) 23 women; 7 men	BSc I and III year students (15) 13 I year; 2 III year
			6 students per team	3 students per team
Duration	3 days (6 h per day)		1 week (4 h per day)*	2 weeks (2 h per day)**
Assessment	Implementation of student workshops in the colleges		Informal conversations, self-reported student feedback	Poster presentation at the research institute, self-reported student feedback

Phase I included an undergraduate teacher workshop followed by Phase II where student workshops were conducted at two colleges. * The workshop was split into two sessions of 2 h, in the morning prior to regular classes and in the afternoon after formal college hours; **the workshop was conducted at the end of the formal college hours.

## Exploring Microbial Diversity in the Laboratory

One of the specific goals of this program was to integrate practical laboratory experiments prescribed in the University syllabus into a research project, changing the focus from laboratory techniques to investigating authentic, real-world biological questions (Supplementary Table 1). This program was designed as a short and intense 1- to 2-week workshop focused on exploring bacterial diversity using 16S RNA T-RFLP workflow (Schütte et al., 2008). Similar workflows can be seen in successful programs such as Small World Initiative and Tiny Earth (Basalla et al., 2020), and Urban Barcode Project (Henter et al., 2016). Students at both colleges worked in teams to explore bacterial diversity in an environmental sample of their choice. They were required to provide reasons for their choice of samples brought for analysis; the samples included raw unpasteurized milk, spoiled fruits and vegetables, cockroach gut, water and soil from a lake and a polluted local river (Musi), iron rust, compost pit, cow dung and effluent from a local chemical industry. All microbial cultures were handled under aseptic conditions and proper lab safety measures were ensured starting from inoculation of cultures to their eventual disposal by autoclaving; safety considerations were discussed before each step of the workflow was executed. After practicing the micropipetting technique and making serial dilutions, students streaked the samples on LB media plates to isolate single bacterial colonies and use those single colonies to perform PCR amplification of the 16S rRNA gene locus using Universal primers (27F and 1492R; Frank et al., 2008). PCR amplicons thus obtained were analyzed using either agarose or polyacrylamide gel electrophoresis (PAGE) before and after digestion with restriction endonucleases such as AluI, DpnI, MboI, and TaqI, which are frequent (4 bp) cutters. The banding patterns corresponding to different bacterial colonies were compared to infer diversity. A brief introduction to genome databases and bioinformatics tools enabling sequence analysis was also given toward the end of the program. *In silico* restriction digestion of 16S rRNA gene sequences from different bacterial species using NEB/web cutter^1^ allowed the students to compare their experimental results with computational analysis. This workflow allowed us to cover several molecular methods and techniques that undergraduate microbiology students benefit from learning and are part of the prescribed syllabus.

Despite the short duration, students were not only able to go through the complete workflow but were able to collect data and document their findings. The collected data included geographical location and nature of samples, serial dilutions and the corresponding density of bacterial colonies on media plates, presence of PCR amplified 16S rRNA gene fragment after gel electrophoresis, observed RFLP patterns, and simulated RFLP patterns using gene sequences from genomic databases. Documentation of their findings became especially important since students had to present the summary of their work at the conclusion of the activity.

Alongside the content of the student activity, several pedagogical considerations were deliberated during the teacher workshop and implemented in the colleges. We made teamwork an essential component so as to enable peer interactions and enhance peer learning. Moreover, the entire activity was based on experiential, hands-on laboratory work beginning from sample collection, planning and executing individual steps of the experiments, and ending with comparing and discussing the results. Therefore, student interactions became essential for successful completion of the experiments and thus were ensured. During the course of the laboratory work, we emphasized on the process of science alongside the execution of instructions and protocols. Questions regarding the underlying principles and logical reasons behind various steps in the experimental protocol were raised at appropriate times.

## Impact of the Student Workshops on Student Learning

To understand and assess the impact of this program, we collected self-reported student feedback at the end of the workshop using a questionnaire with response options in the Likert-scale and open-ended questions. The questions aimed at understanding students’ views on different aspects of the curriculum, teaching and learning practices, and gain of cognitive skills, both in their regular college courses and the workshop. Another set of first- and second-year students who did not attend the workshops were given a similar set of questions focusing only on their college experiences, and their anonymous responses were used as “controls.” A total of 41 students from the two colleges who participated in the workshops (91% response rate) and 102 students from the “control” group completed the questionnaire.

**FIGURE 1:**
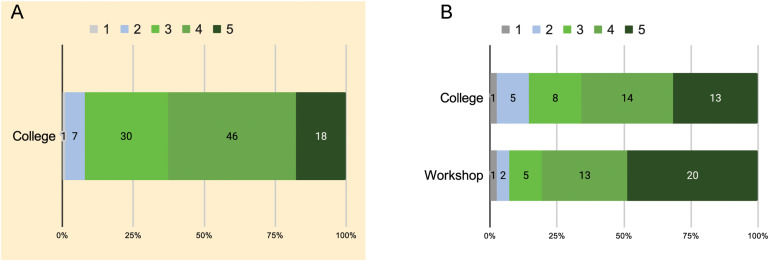
Stacked bar graph showing student rating the impact of college courses and “Exploring Microbial Diversity” workshop on their research and scientific attitudes and skills. **(A)** Rating of college experiences by students who did not participate in the workshop (control) and **(B)** rating of college and workshop experiences by student participants. 1 corresponds to the lowest rating and 5 to the highest rating.

With regards to rating the impact of college courses and the workshop on academic experiences, especially developing science identity and process skills, there is a clear distinction in the number of students who gave a rating of “5” (highest) attributed to regular courses/classes and the “Exploring Microbial Diversity” workshop; 49% of the workshop participants rated the workshop experience at “5”; 31.7% of the workshop participants rated the impact of their college experience at “5”; only 17.65% of the students in the control group rated the impact of college courses at “5” (Figure 1).

The questions focused on three broad categories of students’ learning experiences; key questions from each category included (a) Emphasis, how much either the college or the workshop emphasized on memorization, analysis, synthesis and application (Supplementary Figure 1); (b) Impact, how either the college or the workshop helped them to think independently and analyze concepts (beyond memorization), actively engaged them with academic learning, enhancing their interest in the subject, and gave them full autonomy to plan and execute lab experiments (Supplementary Figure 2); and (c) Involvement, how well they prepared for regular college courses and for the workshop by reading study material, writing extensive notes, reading beyond the prescribed material, and discussing course material with classmates (Supplementary Figure 3). The analysis of all the responses generally indicated that the students gained more from the workshop experience, when compared to their experiences in regular classes. However, the responses do not allow us to draw specific conclusions with regards to differences between student “involvement” with various activities (Supplementary Figure 3).

When asked to comment (as free responses) on the differences they noticed between regular college classes and the workshop, students felt that the workshop enabled them to learn things which they were not able to do and analyze in regular courses, made them to ask questions, think independently, understand concepts better and to apply them effectively. Many students mentioned that they would look forward to more such workshops during their undergraduate studies. One student comment captures the essence of many of these responses – “*Without doubt the “Exploring Microbial Diversity” workshop was better than classes as it involved doing something we do not usually do in classes. Practical experience is the best teacher. But I think a little emphasis on research papers for reference should be included as part of the [laboratory] practicals, and if possible, in classes, as that will not only intensify the research component of the subject but also will give fundamental exposure to scientific literature.*” An analysis of the responses represented in the stacked bar graphs, in conjunction with the free responses, suggests that the workshop experience had a positive impact on most of the students with respect to their engagement with discipline-specific content and also science process skills.

## An Outside Perspective on Student Performance

Students from one of the colleges (Table 1) had an opportunity to present their work to scientists (principal investigators, postdoctoral researchers, graduate students and research associates) at the research institute. This was the only interaction they had with the students. Judging from the presentations, in spite of the variation in the quality, the scientists felt that the students were well prepared, interested, enthusiastic, and demonstrated an ability to understand and critically analyze primary research and experimental findings.

## Teacher Perspectives

The teachers were aware of the limitations and shortcomings related to both material and time resources. Nevertheless, they were encouraged by the fact that we were able to create and implement these pilot programs to engage students, give them an opportunity to explore research questions and enable them to gain deeper insights into real-world microbiology. The key learnings from the first implementation of the “Exploring Microbial Diversity” workshops are that material limitations can be overcome with creative program design in collaboration with the research institute, and time limitations can be overcome by scheduling the programs around regular college hours.

## Discussion

There are several well documented microbiology education programs aimed at providing authentic experiences to undergraduate students (Jordan et al., 2014; Henter et al., 2016; Staub et al., 2016; Basalla et al., 2020). However, in India there are strong perceptions about the difficulty of implementing such programs to systematically improve undergraduate programs. These perceptions are strengthened in the light of poor infrastructure and inadequate facilities in colleges, and time constraints imposed by rigid curricular outcomes. Most of these challenges are likely to be common and widespread in similar academic settings, therefore we need to examine practical ways to overcome these challenges by creating focused college faculty development programs aimed at fostering collaborative programs with research institutes by way of sharing of their resources.

The pilot study described here provides a model for redesigning undergraduate microbiology education in resource-limited environments such as small, private, autonomous, University-affiliated colleges in India. The program focused on inquiry and problem solving in an environment that fosters teamwork and collaboration amongst students. In an academic system rife with intense competition, this is an alternative to expose students to working in teams and enhancing their learning experiences. It is vital that any of these efforts aimed at improving undergraduate STEM education should include rigorous designs and assessments that will help us evaluate learning outcomes of students and thereby the effectiveness of programs (Linn et al., 2015).

The success of high-standard abbreviated “nanocourses” that provide high-impact learning experiences to students (Bentley et al., 2008) provide templates upon which shorter, workshop-style courses can be developed and implemented that address the constraints of time and material resources. Scaffolding authentic research across the curriculum (Sieg et al., 2019) by way of short workshops can be an effective way to leverage academic autonomy and overcome the said constraints. With either internal support from college administration or external support through collaboration with research institutes, teachers can design workshops that cater to the knowledge and skills needs of students while spreading their efforts to implement these workshops. If limited physical presence in colleges is imposed, lecture/discussion-based instruction can happen using synchronous or asynchronous online platforms, while the in-person time can be spent in laboratories to gain important hands-on skills. Scientists in research laboratories are also likely to get involved and collaborate with college teachers to organize such short student programs.

The most significant factor that made this pilot study feasible is the autonomy (even in the limited sense) the participating colleges had in terms of creating and implementing small programs, which allowed the undergraduate instructors to engage in professional development at a research institute. The research institute’s participation was rooted in their interest in ensuring that these students who are prospective recruits are well trained to fuel their future scientific research programs. The active collaboration between research institutes like Cold Spring Harbor Laboratory, Howard Hughes Medical Institute’s (HHMI) Janelia Farms, and undergraduate teaching institutes have given rise to exemplary educational innovations. Similar partnerships, on a smaller scale, can be built and nurtured to support educational activities in local undergraduate institutions. In fact, these smaller partnerships are likely to be stronger and become successful because of greater connectedness. In India, research laboratories belonging to the Council for Scientific and Industrial Research (CSIR), Department of Biotechnology (DBT), Indian Council for Medical Research (ICMR), Indian Council for Agricultural Research (ICAR) are spread across the country and have a wide variety of microbiology-related research programs, which can provide modest support through knowledge- and resource-sharing. This support can result in deep and far-reaching impact on the quality of undergraduate microbiology education.

As long as colleges are willing to find creative ways to use even a limited amount of autonomy at their disposal to innovate in their classrooms, and research institutions with greater resources at their disposal are willing to become enthusiastic supporters and enablers of high-quality educational experiences, there is immense opportunity for students to engage in authentic learning experiences.

## Conclusion

With India unveiling a new National Education Policy in 2020, there is a promise to revamp the entire education landscape in the country. As alluded to earlier, while policies do have a top-down influence on the system, true change is possible when the teachers and colleges are developing and practicing exemplary work at the grassroots. The collaborative model showcased here has given the necessary impetus to the teachers to continue providing rich authentic, real-world microbiology experiences to undergraduate students. We hope that our modest effort and its positive impact on student experiences will inspire and motivate several such local initiatives in resource-limited settings.

The “Vision and Change in Undergraduate Biology Education: A Call to Change” document has recommendations about utilizing innovative pedagogy and the integration of authentic research experiences into individual courses and biology programs overall to ensure that all undergraduates can experience the processes, nature, and limits of science. However, there are several undergraduate institutions with limited resources that are unlikely to provide such experiences to their students. Our Perspective article showcases a collaborative model where a research institute collaborated with two teaching colleges to devise and implement a small program that was effective in providing an authentic research experience to undergraduate students that can be sustainable. The model can be used by other undergraduate institutions with either perceived or real resource limitations.

## Data Availability Statement

The raw data supporting the conclusions of this article will be made available by the authors, without undue reservation.

## Ethics Statement

The studies involving human participants were reviewed and approved by Research Committees of Bhavan’s Vivekananda College and St.Ann’s College for Women. The patients/participants provided their written informed consent to participate in this study.

## Author Contributions

AKC designed the programs and study. YA and KA contributed equally to the implementation of the program. YA, KA, ChJ, KSM, and AKC contributed toward preparation of the manuscript.

## Conflict of Interest

The authors declare that the research was conducted in the absence of any commercial or financial relationships that could be construed as a potential conflict of interest.

## Funding and Acknowledgments

The programs were supported by the Center for Advancement of Research Skills, Dr. Reddy’s Institute of Life Sciences (DRILS). Bhavan’s Vivekananda College was supported by the Star College Scheme, Department of Biotechnology, Government of India (BT/HRD/11/034/2019). AKC thanks Dr. Kiranam Chatti (DRILS), Dr. Madhavan Narayanan (Benedictine University), Dr. Uma Swamy (Florida International University) and Dr. Sanjay Singh (UT MD Anderson Cancer Center) for discussions and feedback on the manuscript. KA and YA thank the members of the Department of Microbiology, Management and Principle of Bhavan’s Vivekananda College. ChJ thanks the members of the Department of Microbiology, Administration and Principal of St. Ann’s College for Women.

## Supplementary Material

The Supplementary Material for this article can be found online at: https://www.frontiersin.org/articles/10.3389/fmicb.2020.589405/full#supplementary-material

Supplementary Figures 1–3 Summary of student responses to the questionnaire. The left panels (in cream box) represents data from the control group (*n* = 102). The second and third columns correspond to responses we obtained from students who participated in the “Exploring Microbial Diversity” workshop (41/45 students completed the questionnaire, 91% response rate); second column corresponds to student/workshop participants experiences in regular college courses, while the third column corresponds to their experiences in the workshop. All data are represented as percentages. Student responses relating to emphasis on memorization, analysis, synthesis, and application (Supplementary Figure 1); impact of college courses or workshop on independent thinking, analysis (beyond memorization), active engagement, enhancement of academic interest, and academic autonomy (Supplementary Figure 2); student involvement with content (Supplementary Figure 3).

## Footnotes

1 http://nc2.neb.com/NEBcutter2/

## References

[B1] Alberts B. (2005). A wakeup call for science faculty. *Cell* 123 739–741. 10.1016/j.cell.2005.11.014 16325564

[B2] Basalla J. Harris R. Burgess E. Zeedyk N. Wildschutte H. (2020). Expanding Tiny Earth to genomics: a bioinformatics approach for an undergraduate class to characterize antagonistic strains. *FEMS Microbiol. Lett.* 367 10.1093/femsle/fnaa018 31971561 PMC8204652

[B3] Bentley A. M. Artavanis-Tsakonas S. Stanford J. S. (2008). Nanocourses: a short course format as an educational tool in a biological sciences graduate curriculum. *CBE—Life Sci. Educ.* 7 175–183. 10.1187/cbe.07-07-0049 18519608 PMC2424306

[B4] Ferren A. S. Anderson C. B. (2016). Integrative learning: making liberal education purposeful, personal, and practical. *New Dir. Teach. Learn.* 2016 33–40. 10.1002/tl.20172

[B5] Frank J. A. Reich C. I. Sharma S. Weisbaum J. S. Wilson B. A. Olsen G. J. (2008). Critical Evaluation of Two Primers Commonly Used for Amplification of Bacterial 16S rRNA Genes. *Appl. Environ. Microbiol.* 74 2461–2470. 10.1128/AEM.02272-07 18296538 PMC2293150

[B6] Frantz K. J. Demetrikopoulos M. K. Britner S. L. Carruth L. L. Williams B. A. Pecore J. L. (2017). A Comparison of Internal dispositions and career trajectories after collaborative versus apprenticed research experiences for undergraduates. *CBE Life Sci. Educ.* 16:206. 10.1187/cbe.16-06-0206 28130268 PMC5332035

[B7] Government of India and University Grants Commission [UGC] (2019). *University Grants Commission: Annual Reports/Annual Accounts, 2018–19.* Available online at: https://www.ugc.ac.in/page/Annual-Report.aspx (accessed July 26, 2020).

[B8] Hatcher J. A. (2011). Assessing civic knowledge and engagement. *New Dir. Institutional Res.* 2011 81–92. 10.1002/ir.382

[B9] Henter H. J. Imondi R. James K. Spencer D. Steinke D. (2016). DNA barcoding in diverse educational settings: five case studies. *Philos. Trans. R. Soc. B Biol. Sci.* 371:20150340. 10.1098/rstb.2015.0340 27481792 PMC4971192

[B10] Jordan T. C. Burnett S. H. Carson S. Caruso S. M. Clase K. DeJong R. J. (2014). A broadly implementable research course in phage discovery and genomics for first-year undergraduate students. *mBio* 5:e01051-13. 10.1128/mBio.01051-13 24496795 PMC3950523

[B11] Lakhotia S. C. Shashidha L. S. Vale R. (2013). Excellence in science education and research. *Curr. Sci.* 104 163–165.

[B12] Linn M. C. Palmer E. Baranger A. Gerard E. Stone E. (2015). Undergraduate research experiences: Impacts and opportunities. *Science* 347:1261757. 10.1126/science.1261757 25657254

[B13] Association of American Colleges and Universities (2007). *College Learningfor the New Global Century A Report from the National Leadership Council for Liberal Education & America’s Promise.* Washington, DC: Association of American Colleges and Universities.

[B14] Penn J. D. (2011). The case for assessing complex general education student learning outcomes. *New Dir. Institutional Res.* 2011 5–14. 10.1002/ir.376

[B15] Saberwal G. (2019). Will our education system enable India to be a super-power any time soon? *Curr. Sci.* 116 509–510.

[B16] Schütte U. M. E. Abdo Z. Bent S. J. Shyu C. Williams C. J. Pierson J. D. (2008). Advances in the use of terminal restriction fragment length polymorphism (T-RFLP) analysis of 16S rRNA genes to characterize microbial communities. *Appl. Microbiol. Biotechnol.* 80 365–380. 10.1007/s00253-008-1565-4 18648804

[B17] Sieg R. D. Narayanan M. Sabatini J. Beverly N. Surendran G. Smyth D. S. (2019). Incubating the SENCER ideals with project-based learning and undergraduate research: perspectives from two liberal arts institutions. *Sci. Educ. Civ. Engagem.* 11 50–63.

[B18] Staub N. L. Poxleitner M. Braley A. Smith-Flores H. Pribbenow C. M. Jaworski L. (2016). Scaling up: adapting a phage-hunting course to increase participation of first-year students in research. *CBE—Life Sci. Educ.* 15:ar13. 10.1187/cbe.15-10-0211 27146160 PMC4909335

[B19] Wei C. A. Woodin T. (2011). Undergraduate research experiences in biology: alternatives to the apprenticeship model. *CBE—Life Sci. Educ.* 10 123–131. 10.1187/cbe.11-03-0028 21633057 PMC3105915

[B20] Zai R. (2015). Reframing general education. *J. Gen. Educ.* 64 196–217. 10.5325/jgeneeduc.64.3.0196

