# Supplementary figures and images for: Bringing Real-World Microbiology Experiences to Undergraduate Students in Resource-Limited Environments
Source: Front Microbiol. 2020 Dec 8;11:589405. doi: 10.3389/fmicb.2020.589405 (PMC7752863; doi:10.3389/fmicb.2020.589405)

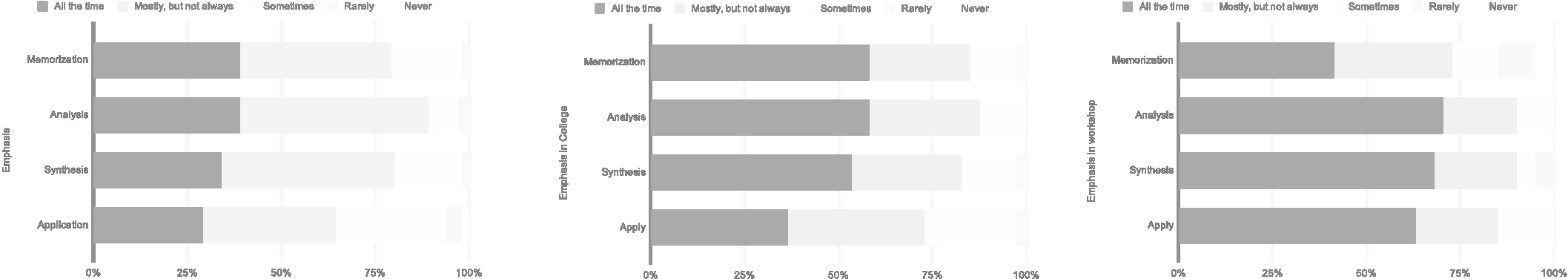

Supplement: Supplementary Figures 1–3 — Summary of student responses to the questionnaire. The left panels (in cream box) represents data from the control group (n = 102). The second and third columns correspond to responses we obtained from students who participated in the “Exploring Microbial Diversity” workshop (41/45 students completed the questionnaire, 91% response rate); second column corresponds to student/workshop participants experiences in regular college courses, while the third column corresponds to their experiences in the workshop. All data are represented as percentages. Student responses relating to emphasis on memorization, analysis, synthesis, and application (Supplementary Figure 1); impact of college courses or workshop on independent thinking, analysis (beyond memorization), active engagement, enhancement of academic interest, and academic autonomy (Supplementary Figure 2); student involvement with content (Supplementary Figure 3). [file Image_1.tiff]

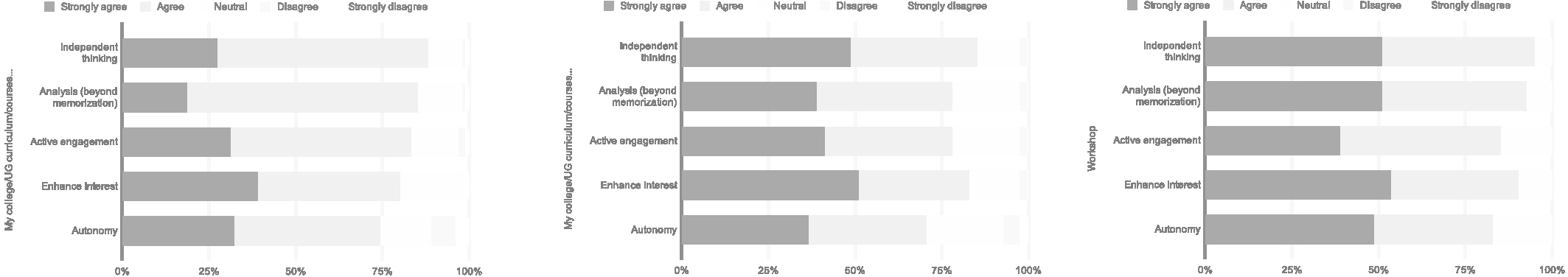

Supplement: Supplementary file 2 [file Image_2.tiff]

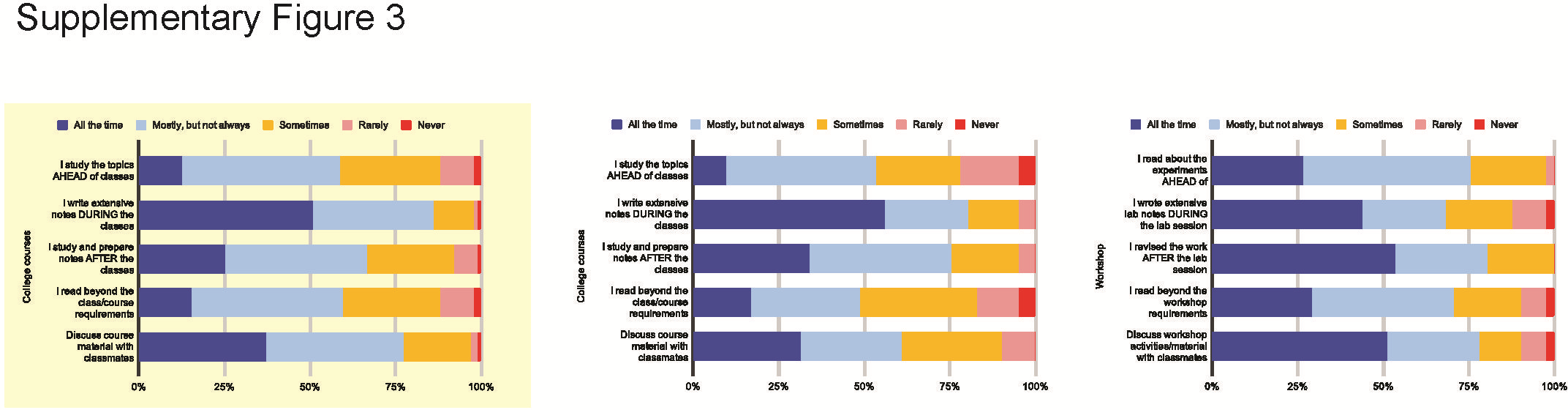

Supplement: Supplementary file 3 [file Image_3.tiff]
