# Supplementary material for: Bringing Real-World Microbiology Experiences to Undergraduate Students in Resource-Limited Environments
Source: Front Microbiol. 2020 Dec 8;11:589405. doi: 10.3389/fmicb.2020.589405 (PMC7752863; doi:10.3389/fmicb.2020.589405)
Supplement: Supplementary file 4 [file Data_Sheet_1.PDF]

Supplementary Table 1: Student Learning Objectives and corresponding Bloom's Level suggestions

	Student Learning Objectives	Suggested Bloom's Level
**Conceptual Knowledge**		
1.	Estimate and evaluate the bacterial load of collected environmental samples	Evaluation
2.	Compare and contrast morphological and molecular identification methods of bacterial species	Evaluation
3.	Identify and analyze diversity of bacterial species in different environmental samples by analyzing DNA sequences	Comprehension
**Laboratory Skills**		
1.	Plan and collect environmental samples	Application and Synthesis
2.	Perform serial dilution	Comprehension and Synthesis
3.	Plate and streak bacterial samples	Comprehension and Synthesis
4.	Setting up Polymerase Chain Reaction using individual bacterial colonies	Comprehension and Synthesis
5.	Setting up restriction endonuclease digestion reactions	Comprehension and Synthesis
6.	Preparing polyacrylamide gels (Explain the chemistry of acrylamide:bis-acrylamide polymerization)	Comprehension and Synthesis
7.	Perform gel electrophoresis to separate DNA fragments	Comprehension and Synthesis

8.	Image DNA gels and visualize DNA fragments	Comprehension and Synthesis
9.	Search and obtain 16S rRNA gene sequences using ENSEMBL	Comprehension and Synthesis
10.	Perform in-silico digestion of 16S rRNA sequences using NEBCutter/WebCutter	Comprehension and Synthesis
11.	Setting up Polymerase Chain Reaction using individual bacterial colonies	Comprehension and Synthesis
